# Dietary Acid Load and Relationship with Albuminuria and Glomerular Filtration Rate in Individuals with Chronic Kidney Disease at Predialysis State

**DOI:** 10.3390/nu14010170

**Published:** 2021-12-30

**Authors:** Luísa Silva, Sara Alegria Moço, Maria Luz Antunes, Andreia Sousa Ferreira, Ana Catarina Moreira

**Affiliations:** 1 Nutrition and Dietetics Department, Beatriz Ângelo Hospital, 2674-514 Loures, Portugal; sara.moco@hbeatrizangelo.pt (S.A.M.); andreia.ferreira@hbeatrizangelo.pt (A.S.F.); 2 ESTeSL—Escola Superior de Tecnologia da Saúde de Lisboa, Instituto Politécnico de Lisboa, 1990-096 Lisboa, Portugal; mluz.antunes@estesl.ipl.pt (M.L.A.); ana.moreira@estesl.ipl.pt (A.C.M.); 3 Faculdade de Medicina, Universidade de Lisboa, 1649-004 Lisboa, Portugal; 4 APPsyCI—ISPA, 1149-041 Lisboa, Portugal; 5 H&TRC—Health and Technology Research Center, 1990-096 Lisboa, Portugal

**Keywords:** chronic renal insufficiency, acidosis, diet

## Abstract

The Western diet, characterized by excessive consumption of animal protein and reduced intake of vegetables and fruits, is also rich in sulfur, chlorine, and organic acids, which are the main sources of dietary acid load. A relationship between dietary acid load, renal function, and progression of chronic kidney disease has been demonstrated. Dietary modifications seem to contribute to a reduction in dietary acid load, and are associated with improved outcomes in individuals with chronic kidney disease (CKD). The aim of this paper was to review the existing evidence concerning the association between dietary acid load and renal function in nondialyzed individuals with CKD. A systematic review was conducted by gathering articles in electronic databases (MEDLINE/PubMed, Scopus, and Web of Science) from January 2018 to May 2021. Dietary acid load and GFR and/or albuminuria were analyzed. A total of 1078 articles were extracted, of which 5 met the inclusion criteria. Only one study found no statistically significant associations between the study variables. The remaining showed a negative association between dietary acid load and renal function. This systematic review confirmed the existence of an association between dietary acid load and renal function, with a high dietary acid load contributing to a decreased renal function.

## 1. Introduction

Chronic kidney disease (CKD) is a widespread public health problem throughout the Western world, and is growing in the community [1]. This condition affects about 10% of the world’s population. There are several risk factors associated with CKD, with diabetes being the main one, followed by hypertension (HT) and glomerulonephritis. The first two are responsible for 60% of the aetiology of this disease. About 1 in 3 people with diabetes and 1 in 5 people with hypertension have CKD. Cardiovascular disease and obesity are, together with the above, important diet-related risk factors that can condition the progression of CKD [2,3].

The estimated prevalence of CKD worldwide is 9.1%, contributing to 1.2 million deaths in 2017. According to the 2017 Global Burden of Disease Study, CKD is the 12th leading cause of death, out of 133 diseases [4]. The prevalence of CKD at stages 1–3 is 1.29 times higher in females than in males. On the other hand, mortality is 1.39 times higher in males, suggesting that men progress more rapidly to more advanced stages of CKD [4].

Diet is considered the most important individual factor affecting individual’s acid–base status. Typical Western diets, rich in protein of animal origin, provide about 1mmol/kg body weight/day of endogenous excretion of H+, mainly due to the metabolism of sulfur-rich amino acids (methionine and cysteine). On the other hand, food constituents that are precursors of bases are mostly of plant origin (involves the metabolism of organic anions such as citrate and malate). Total acid excretion is analytically quantified by the 24-h urine collection, whereby the urinary excretion of ammonia is quantified. The analytical determination of total acid excretion reflects the renal acid load in healthy individuals [5].

On the other hand, total urinary acid excretion can be reasonably estimated through dietary intake, intestinal absorption, and urinary metabolism of major inorganic anions and cations. Taking these observations into account, Remer and Manz developed a model known as the Renal Acid Load Potential (PRAL) [6,7]. With the use of nutrient intake data, this method assesses the acid load produced by the diet. Thus, it is possible to estimate the total acidity of the diet.

This model takes into account the intake of protein, phosphorus, potassium, magnesium, and calcium, and is based on the average intestinal absorption rate of each nutrient and the urinary excretion of organic acids (OA) [6,7]. When the PRAL value for a certain food is below 0, it can be assumed that it increases the alkalinity of body fluids. On the contrary, when it is above 0, the production of acids is potentiated by this food. In general, foods such as meat, eggs, cheese, and cereals increase acid production, while fruits and vegetables enhance the production of alkaline compounds in the body. Milk and milk products, sugar, and fat are considered neutral, since they have a reduced effect on the acid–base balance [6,7,8].

Nutritional therapy is an essential component in the prevention and treatment of CKD, with intervention in the predialysis phase focusing on the reduction of some nutrients such as protein, phosphorus, and sodium [9,10]. These dietary modifications, recommended by current guidelines, lead to a reduction in dietary acid load (DAL) and are associated with improved outcomes in individuals with CKD [9,11]. 

Since acid–base homeostasis is maintained through urinary acidification, as renal function degrades, the need for acidification by the residual nephrons increases. This leads to an increase in ammonia production, partly controlled by endothelin, causing injury to the residual nephrons [12]. Acid retention also has the potential to promote muscle degradation as part of the homeostatic process to normalize the acid–base balance. Metabolic acidosis increases proteolysis in skeletal muscle, contributing to an adverse impact on the patient’s nutritional status. Based on these mechanisms, individuals with CKD stages 1 to 4 should follow the KDOQI guidelines which suggest reducing endogenous acid production (NEAP) through dietary intake of fruit and vegetables to slow down the rate of residual renal function decline [13].

The aim of this paper was to conduct a systematic review to analyze possible associations between dietary acid load and renal function. There is already a systematic review with a meta-analysis on the same topic, but which only included observational study articles published up to 2018. Since there is high scientific production on the subject of kidney disease, we expected that studies published in recent years would corroborate or refute this association.

## 2. Materials and Methods

### 2.1. Protocol and Registration

The protocol for this systematic review was registered in the International Prospective Register of Systematic Reviews (PROSPERO) under the registration number CRD42021270640.

### 2.2. Search Strategy

A systematic literature search was conducted using the Preferred Reporting Items for Systematic Reviews and Meta-Analyses (PRISMA) checklist [14] to assess the updated scientific evidence concerning the association between dietary acid load and renal function in nondialyzed CKD patients.

The latest systematic review on this topic data was published in 2018, thus, articles published between January 2018 and May 2021 in the MEDLINE/PubMed, Scopus and Web of Science databases were considered. 

The search query was divided into 2 parts and included the MeSH terms chronic renal insufficiency, chronic kidney failure, kidney diseases, renal insufficiency, albuminuria, proteinuria, glomerular filtration rate, acid base imbalance, metabolic acidosis, Western diet, high-protein diet and plant-base diet. Non-MeSH terms such as CKI, CRF, ESRD, CKD, serum creatinine, serum albumin, microalbuminuria, urine albumin, serum bicarbonate, urine albumin excretion rate, urine protein, urine protein excretion, urine albumin to creatinine ratio, urine creatinine, urine albumin, serum pH, dietary acid load, potential renal acid load, net endogenous acid production, net acid excretion, gastrointestinal alkali absorption, were also included.

### 2.3. Study Selection and Eligibility Criteria

Regarding the inclusion criteria, studies with the following criteria were selected: Original studies; articles published in Portuguese (the authors, natural language) and English; studies published between 1 January 2018 and 30 May 2021; studies containing the calculation of dietary acid load (obtained by PRAL and/or NAE) and simultaneously calculation of renal function (albuminuria and/or GFR); studies with adult individuals with chronic renal insufficiency; and studies conducted in adults (age ≥18 years).

Studies with the following characteristics were excluded: articles such as commentaries, letters to the editor, reviews, conference proceedings, opinion articles; studies that did not present results or prevalence; studies carried out in pregnant women; and studies in patients undergoing renal function replacement therapy.

### 2.4. Risk of Bias Assessment

The evaluation of the articles was carried out independently by two researchers (L.S. and A.F.). Articles in which there was no consensus between the two investigators on whether to include or exclude them were discussed with a third investigator (A.C.M.). Analysis of the articles was performed using the Rayyan software. 

Full-text included articles were assessed for their methodological quality using the Newcastle–Ottawa scale [15]. In this systematic review, studies with scores higher than 6 were considered as high-quality studies.

## 3. Results

Initially, 1409 articles were identified; 435 in PubMed, 595 in Scopus, and 378 in Web of Science, of which 329 were duplicates. After assessment of title and abstract, 1065 articles were excluded for being review papers, animal studies, studies conducted with children, and irrelevant studies regarding the topic (studies that did not assess CKD or markers that did not include the defined outcomes). In the end, the eligibility of 12 articles gathered was assessed by reading them in full. After exclusion of seven publications, five articles were finally included in the systematic review. Of the seven excluded articles, two were written in a language other than English or Portuguese, two assessed variables other than those defined, and three were excluded for not fitting the type of study criterion.

**Figure 1:**
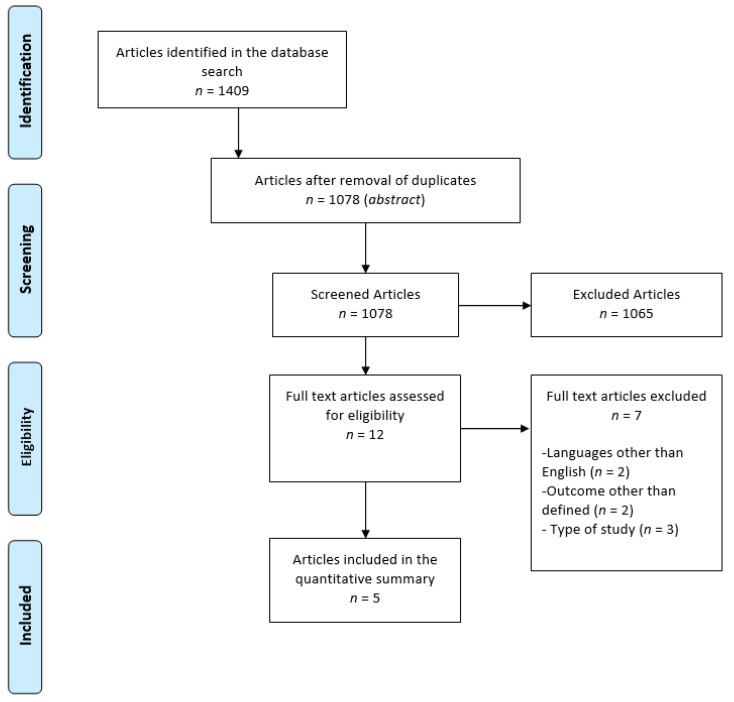
Diagram for the selection of articles.

The organigram for the selection of articles is shown in Figure 1, and the results are described in Table 1. The total scores regarding the methodological quality of the analyzed articles are presented in Table 1 and partial scores can be found in supplementary material Table S1.

The included studies were published between 2018 and 2019 and were conducted in the United States of America (USA) (*n* = 3) or Japan (*n* = 2). All assessed the association of dietary acid load (DAL) in individuals of both genders in samples ranging from 95 to 6684 individuals. DAL was assessed using PRAL (*n* = 3), NEAP (*n* = 3), or NAE (*n* = 2), with three studies using both PRAL and NEAP [16,17,18,19].

In 2019, Pike and colleagues [19] conducted a prospective observational study aiming to assess possible associations between metabolic acidosis or dietary acid load and CKD progression according to APOL1 genotype. This study had a mean follow-up period of 7 years, evaluating 1048 African American participants. Regarding the intervention, dietary acid load was calculated by applying a Food Frequency Questionnaire (FFQ) validated for the study population, and subsequent calculation of PRAL (by the Remer and Manz equation) [6,7] and NEAP (Frassetto equation) [21,22]. Participants were divided into quartiles according to NEAP, and the outcomes defined were GFR and development of acute kidney injury. After adjustment for confounding variables, no associations were detected between dietary acid load and kidney disease progression in any APOL1 genotype (HR, 0.98; 95% CI, 0.93–1.04 and HR, 1.03; 0.95% CI, 0.92–1.15).

Similar to the previous study, in 2018, Banerjee and colleagues [17] also assessed a sample of African American individuals (3275 subjects) included in a cross-sectional cohort study that ran from 2000 to 2004. Dietary intake was assessed using a validated FFQ for the study population, and dietary acid load was calculated using the PRAL and urinary acid excretion equation [6,7]. Subjects were divided into tertiles according to dietary acid load. Primary outcome variables were reduced renal function defined by GFR and albuminuria. Among participants with hypertensive nephropathy, it was further explored whether endothelin and aldosterone production mediated the association between DAL and GFR. After adjustment for potential confounders, although the highest tertile of DAL showed 1.2 times more likelihood of having albuminuria (OR 1.15; 95% CI: 0.75–1.70), this association was not statistically significant. Only after multivariable analysis with adjustment for confounders, the highest and the middle tertile of DAL was found to be almost three times more likely to have reduced renal function compared with the lowest tertile (OR 2.82; 95% CI: 1.40–4.75). A higher DAL was still statistically associated with reduced renal function, even after adjustment for potential confounders (*p* = 0.02). Finally, in the final models adjusted for aldosterone and endothelin, the statistical significance of the association between dietary acid load and albuminuria was attenuated.

In 2019, Kabasawa and colleagues [18] conducted a cross-sectional cohort study with 6684 residents of the Uonuma region in Japan, including data from 2012 to 2015. Regarding outcomes, GFR and albuminuria were analyzed, establishing cutoff values to define three albuminuria intervals. Dietary acid load was calculated based on a validated FFQ for the study population and PRAL and NEAP equations [6,7], both analyzed in quartiles. The association between NEAP quartiles and the three levels of albuminuria was analyzed: regarding the presence of microalbuminuria, the highest quartile of NEAP was associated with a higher risk (odds ratio) in men (OR 1.47; 95% CI: 1.08–1.99; *p* = 0.0130) and women (OR 1.54; 95% CI: 1.11–2.14; *p* = 0.0014) in the fully adjusted model; regarding the presence of high albuminuria, the highest NEAP quartile was associated with a higher risk (odds ratio) in women (OR 1.34; 95% CI: 1.05–1.70; *p* = 0.0163), but not in men (OR 1.18; 95% CI: 0.93–1.51; *p* = 0.2391); finally, regarding the presence of elevated albuminuria or microalbuminuria, the highest quartile of NEAP was associated with a higher risk (odds ratio) in men (OR 1.28; 95% CI: 1.02–1.59; *p* = 0.0407) and women (OR 1.39; 95% CI: 1.11–1.74; *p* = 0.0028). An analysis was also performed regarding the nutrients found to be associated with NEAP and albuminuria, with an association between the highest quartile of potassium intake and lower risk (odds ratio) for microalbuminuria. The odds ratio adjustment for the presence of elevated albuminuria or microalbuminuria comparing the highest and lowest quartile of potassium intake was 0.73 (95% CI: 0.57–0.94, *p* = 0.0094) for men and 0.75 (95% CI: 0.59–0.95, *p* = 0.0304) for women. In summary, there was a negative relationship between potassium intake in early stages of albuminuria and DAL.

Toba and colleagues [16] conducted a cross-sectional cohort study published in 2019 with data from 95 patients followed at Niigata University Hospital in Japan from 2008 to 2014 to investigate the association between dietary acid load and CKD progression. They also analyzed which types of food significantly affected DAL. A validated FFQ for the study population was used to calculate DAL, and then the Frassetto equation was used to estimate NEAP [22], and patients were categorized into two groups based on the mean NEAP value. As for the outcome, the GFR was calculated. The mean GFR was slightly higher in individuals with a higher NEAP when compared with individuals with lower NEAP. A significant interaction (*p* = 0.035) was observed between time and NEAP, showing that the decline in mean GFR was significantly higher in individuals with higher NEAP. Analysis using logistic regression revealed that low fruit (adjusted OR, 6.45; 95% CI, 2.19–19.00; *p* = 0.001) and vegetable (adjusted OR, 3.87; 95% CI, 1.29 – 11.6; *p* = 0.016) intake was significantly associated with high NEAP, as opposed to high red meat intake, which was associated with higher NEAP (adjusted OR = 2.64; 95% CI, 0.92–7.61; *p* = 0.071).

In 2019, Brown and colleagues [20] studied a subcohort of the CRIC study. To better understand new paradigms that linked higher acid excretion with better CKD outcomes, they explored possible predictors of NAE. The CRIC study was a prospective observational study of 3939 individuals with CKD, who were recruited between 2003 and 2008 at 13 centers across the USA. A subgroup of 978 American participants with a mean age of 58 years and mean GFR of 44mL/min/1.73m² was assessed while taking into account some clinical, demographic, laboratory, and body-composition characteristics. Regarding the intervention, dietary acid load was measured with NAE, calculated as the sum of urine ammonium and titratable acidity. The authors chose demographic, comorbid conditions, medications, laboratory value predictors (including GFR estimated with the CKD epidemiology collaboration equation), and PRAL (calculated using a diet history questionnaire and using the Remer and Manz equation), which were then evaluated for an association with NAE in unadjusted, minimally adjusted, and fully adjusted models. In the unadjusted associations between predictors and NAE, higher NAE was associated with greater GFR (3.65; 95% CI: 2.58 to 4.71; *p* < 0.001), increasing insulin resistance (2.50; 95% CI: 1.39 to 3.61; *p* < 0.001), and higher PRAL (3.22; 95% CI: 1.96 to 4.48; *p* < 0.001), among others. After multivariable adjustment, insulin resistance (1.51; 95% CI: 0.43 to 2.58; *p* = 0.006), GFR (2.99; 95% CI: 1.95 to 4.02; *p* < 0.001), and PRAL (1.78; 95% CI: 0.62 to 2.94; *p* = 0.003), among others, remained associated with higher NAE. Finally, in the fully adjusted model, higher NAE remained directly associated with greater GFR (3.07; 95% CI: 1.87 to 4.27; *p* < 0.001), higher insulin resistance (1.76; 95% CI: 0.55 to 2.98; *p* = 0.005), and higher PRAL (1.79; 95% CI: 0.56 to 3.03; *p* = 0.004).

## 4. Discussion

This systematic review aimed to update the scientific literature described on the topic of dietary acid load and renal function in nondialyzed individuals, from 2018 to date. The need for an update on this topic arose because in the meantime, articles that included a large number of patients with a high methodological quality were published [23]. Furthermore, this topic is of extreme importance, considering the relevance to clinical practice and nutritional therapy interventions in CKD.

This new data confirmed and brought robustness to the previous associations found between dietary acid load and renal function. A higher dietary acid load appears to be associated with a decline in kidney function, and according to Toba and colleagues [16], this association was attributed to a low intake of vegetables and fruits. In both studies by Kabasawa and Banerjee [17,18], an association was found between dietary acid load and albuminuria, a marker defined in this systematic review for decline in renal function. The demonstrated association between elevated normoalbuminuria and dietary acid load was of note, as the former can lead to adverse outcomes such as cardiovascular disease, end-stage renal disease, and increased mortality. The study by Kabasawa and colleagues [18] further showed that potassium is an important dietary component in the association between dietary acid load and albuminuria. On the other hand, the study by Pike and colleagues [19] could not show a statistically significant relationship between outcomes. Acidosis was associated with CKD progression in the "raw" analysis, but not when adjusted for confounders, as the data lacked statistical power to detect an effect of this magnitude. The authors mentioned as a major limitation the lack of statistical power, since the study lacked power to detect associations of a small magnitude. According to the authors, lower acid excretion may be a reflection of impaired ability of the kidney to excrete ammonia than of a decrease in acid load, a hypothesis that was in line with the results obtained by Brown and colleagues. It should be noted, however, that Pike and colleagues reinforced the idea that dietary acid load is a modifiable risk factor of importance in the therapy of patients at risk for ESRD [19].

As pointed out above, Brown et al. suggested a novel hypothesis, that higher NAE levels were associated with a lower risk for CKD progression in patients with diabetes. Differences between predicted and measured acid load may be due to an incomplete understanding of the determinants of acid excretion. In this study, the authors suggested that differences in basal energy metabolism resulted in acid production independent from diet, with greater impact than diet or kidney function. As pointed out by the authors, in the study, urine specimens were tested after storage of up to 10 years, which may have affected the accuracy of measurements. It is also important to mention that this study used a different calculation methodology for NAE, compared to the other studies presented in this review. Thus, comparison between studies was hampered. The authors stated that more studies with metabolic assessment were necessary to test the new paradigm [20].

The importance of the topic of this systematic review and consequent update for the clinical practice of healthcare professionals in the context of CKD has become increasingly evident. Recent literature expresses the fact that despite multiple therapies, the reduction in GFR remains progressive [13]. Thus, there is a need for further adjuvant interventions to protect kidney functions. Due to these findings, the Kidney Disease Outcomes Quality Initiative Guidelines suggest that increased fruit and vegetable intake may reduce body weight, blood pressure, and endogenous acid production, based on a grade 2C recommendation [13].

The main cause of CKD is diabetic nephropathy, accounting for 30–40% of cases. On the other hand, glumerolonephritis accounts for approximately 15% of CKD cases, and primarily affects younger people [24]. Hypertension is present in approximately 80–85% of individuals with CKD, and can be both a cause and a consequence of CKD [25]. Individuals with CKD, regardless of diagnosis, are at high risk of cardiovascular disease, including coronary heart disease, cerebrovascular disease, peripheral vascular disease, and heart failure. Most individuals with CKD die from complications related to cardiovascular disease, rather than progressing to kidney failure [26]. The clinical characteristics of patients included in the analyzed studies (shown in Table 1) were in line with the prevalence of CKD-related diseases described in the literature. Patients included in the selected studies were representative of the general population. Thus, prevention and treatment of cardiovascular risk factors through nutritional therapy is of utmost importance for CKD control.

Patients with diabetic nephropathy are clinically characterized by a more rapidly decline in renal function over time. According to Sasso and colleagues [24], weight, albumin excretion rate, and sodium excretion reduction are of great importance in blood pressure control in diabetic patients with diabetic nephropathy. The study by Sasso et all also emphasized that type 2 diabetic patients with diabetic nephropathy should receive intensive multifactorial treatment on cardiovascular and renal factors. As pointed out earlier, cardiovascular risk factors in patients with kidney disease led to late complications and worse outcomes. These risk factors are modifiable, and can be prevented/treated with dietary interventions. More recently, Sasso and colleagues [27] carried out a multicenter, cluster-randomized, open-label clinical trial with diabetic kidney disease patients; they concluded that implementing an intensive multifactorial treatment of cardiovascular risk factors is an important approach to significantly reduce the risk of major fatal and nonfatal cardiovascular events in type 2 diabetic kidney disease patients. 

In the context of emerging evidence over recent years, healthy eating patterns that include plant-based foods may not only be safely incorporated into the diet of individuals with CKD, but may also favorably assist in disease management. Unlike drug therapy, dietary-level changes have the potential to address the cause of lifestyle-related diseases for many individuals, and may result in improvement of multiple disease processes simultaneously [28,29].

The calculation methodology based primarily on PRAL allows an appropriate estimation of dietary effects on urine acidity. The NAE calculation has already been validated in healthy adults, and shows that acid load and renal acid excretion can be reliably estimated from diet composition [6,7]. As described by Remer and Manz, animal protein and cereals are considered acid-inducing foods, which are in turn metabolized into acidic waste products. Animal protein has high levels of phosphorus, thus contributing to the acidity of body fluids. We can therefore say, according to the equations of Remer and Manz, that diets rich in protein present a greater acid load.

In addition to having potential implications for kidney function and structure, high-protein diets can lead to other metabolic complications. A high-protein diet can lead to elevated levels of urea and other nitrogenous waste products. As discussed earlier, a high-protein diet can lead to metabolic acidosis in individuals with advanced CKD who already have deficient acid excretion and bicarbonate generation on their own, especially in the context of animal protein.

Our results were consistent with one literature review published in 2020 [30] that found that food-borne acid may be a risk factor for CKD through intrarenal mechanisms that promote kidney injury and progressive decline in GFR. In an animal model of CKD, chronic metabolic acidosis could stimulate the production of angiotensin II, aldosterone, and endothelin-1, as well as ammonia genesis, all of which have the potential to promote inflammation and fibrosis [29].

As described earlier, the standard American diet, in which protein comprises about 15% of energy, produces a dietary acid load of approximately 1 mEq/kg/day [29]. In contrast, by including a greater proportion of naturally alkaline foods such as fruit and vegetables in the diet, the diet can become almost neutral. Plant-based foods can thus be used to reduce both the acid load of the diet and the severity of metabolic acidosis [28,29]. In a randomized controlled trial with 108 individuals with stage III CKD, the administration of 2 to 4 cups of fruit and vegetables per day was comparable with oral sodium bicarbonate intake in increasing serum bicarbonate levels in the treatment of metabolic acidosis after a 3-year period [31]. These results were in line with our findings in this systematic review; for example, the findings of Toba and colleagues regarding the influence of fruit and vegetables on the association between acid load and renal function [16].

Our systematic review used articles based on data from large cohorts in the USA and Japan. The methodology for intake assessment was solid, since the studies applied food frequency questionnaires validated for the populations in question, and thus correctly assessed what was intended. Other strengths of the studies used in our review were the inclusion of confounders in the analysis between renal outcomes. We followed the steps and stages of the PRISMA checklist to reduce the risk of biases associated with the search and analysis of articles.

The limitations of this study cannot be underestimated. The studies used for this systematic review were all observational studies, which did not allow assessing a causal relationship between dietary acid load and renal function. In addition, all the studies used food frequency questionnaires that, although validated, were carried out by the patient themselves, which could have led to errors in their completion: in addition to relying on memory, it is difficult to estimate portion sizes, and recall of previous intake may be influenced by current intake. However, any other method of intake assessment also presents the possibility of bias. Thus, their complexity and length may influence adherence [32]. In almost all studies, the exclusion of individuals during the process of applying inclusion criteria may also have resulted in bias in the studies themselves. With regard to the construction and development of the review itself, studies with languages other than English were excluded, and only three databases were used to search for articles, which may have led to the loss of articles that had the possibility of being included. On the other hand, the databases considered to be more comprehensive were chosen.

Since a few years have passed since the latest review on the topic was carried out, and bearing in mind that the latter included only observational studies, it would be important to check for new studies that would further increase the current knowledge. With our systematic review, we have already included two prospective studies that were better than prospective studies, with patients desirably randomized to different acid load diets, which will reinforce these findings.

## 5. Conclusions

Considering the current evidence, our systematic review gives strength to the previous findings, supported by the analysis of a large number of patients. A high dietary acid load was directly associated with an increased risk of CKD and a decline in kidney function. However, further studies with greater statistical power, such as randomized controlled trials, will establish the causal relationship between these variants. We also proposed further investigation of some questions regarding the mechanisms through which this relationship is mediated, such as the influence of angiotensin II, aldosterone, and endothelin-1, as well as a more detailed study of the effects of different types of protein (vegetable versus animal) on renal function.

In the light of the most recent findings, we can also state that for clinical applicability, calculation of dietary acid load can be easily performed by means of records, data collection, or food frequency questionnaires, and it should be incorporated into the nutritional therapy of the disease. Dietetic intervention with reduced protein content, replaced by vegetable protein and coupled with increased fruit and vegetable intake, can reduce the amount of hydrogen ions in the body, thus improving metabolic parameters of acidosis and reducing kidney damage and disease progression.

The inclusion of dietary acid load in nutritional therapy may be an important strategy for future interventions in populations at risk for CKD, and may contribute to the control of metabolic acidosis.

## Figures and Tables

**Table 1:** Analyzed studies.

Reference	Study Type	Population	*n*	Clinical Characteristics (%)	Intervention (Calculation of)	Results	ES
[16]	Cross-sectional cohort	Patients followed between 2008 and 2014 in Niigata, Japan	95	DM2: 64 HTA: 100	NEAP and GFR	The reduction in mean GFR was significantly greater in patients with a higher NEAP	8
[17]	Cross-sectional cohort	African American patients in the Jacson Heart study, 2000 to 2004 in Mississippi, USA	3275	DM2: 20,6 HT: 61,3 CVD: 7,8	NAE, PRAL, GFR, and ACR	A higher DAL was associated with reduced renal function	8
[18]	Cross-sectional cohort	Participants in the Uonuma Cohort study, 2012 to 2015 in Niigata, Japan	6684	DM2: 6,6 HT: 51,2	NEAP, PRAL, GFR, and ACR	A higher NEAP was associated with a higher ACR; a higher NEAP was associated with a higher OR of microalbuminuria in men and woman	9
[19]	Prospective observational study	African American participants in the CRIC study, 2003 to 2008, USA	1048	DM2: 50 HT: 93,3 CVD: 38	PRAL, NEAP, and GFR	No association between PRAL and CKD progression was found	9
[20]	Cohort	Participants in the CRIC study, 2003 to 2008, USA	978	DM2: 50,7	NAE and GFR	A higher NAE was associated with a higher GFR	9

DM2 = diabetes mellitus 2; HT = hypertension; CVD= cardiovascular disease; NAE = net acid excretion; NEAP = net endogenous acid production; PRAL = potential renal acid load; ACR = albumin/creatinine ratio; DAL = dietary acid load; OR = *odds ratio*; ES = evidence score.

## Data Availability

The protocol for this systematic review was registered in the International Prospective Register of Systematic Reviews (PROSPERO) under the registration number CRD42021270640.

## Supplementary Materials

The following are available online at https://www.mdpi.com/article/10.3390/nu14010170/s1, Table S1: Newcastle Ottawa Quality Assessment Scale.
