# Supplementary material for: Dietary Acid Load and Relationship with Albuminuria and Glomerular Filtration Rate in Individuals with Chronic Kidney Disease at Predialysis State
Source: Nutrients. 2021 Dec 30;14(1):170. doi: 10.3390/nu14010170 (PMC8746801; doi:10.3390/nu14010170)
Supplement: Supplementary file 1 [file nutrients-14-00170-s001.zip › nutrients-1493555-supplementary.pdf]

Table S1. NEWCASTLE–OTTAWA QUALITY ASSESSMENT SCALE

	Selection	Comparability	Outcome	Final Score
(Toba et al. 2019)	4	2	2	8
(Banerjee et al. 2018)	4	2	2	8
(Kabasawa et al. 2019)	4	2	3	9
(Pike et al. 2019)	4	2	3	9
(Brown et al. 2019)	4	2	3	9
